# Clinical trials with cannabis medicines—guidance for ethics committees, governance officers and researchers to streamline ethics applications and ensuring patient safety: considerations from the Australian experience

**DOI:** 10.1186/s13063-020-04862-6

**Published:** 2020-11-17

**Authors:** Jennifer H. Martin, Courtney Hill, Anna Walsh, Daryl Efron, Kaitlyn Taylor, Michael Kennedy, Rachel Galettis, Paul Lightfoot, Julie Hanson, Helen Irving, Meera Agar, Judith Lacey

**Affiliations:** 1 Australian Centre for Cannabinoid Clinical and Research Excellence (ACRE), Division of Clinical Pharmacology, School of Medicine and Public Health, University of Newcastle, Newcastle, New South Wales Australia; 2 NHMRC Clinical Trials Centre, University of Sydney, Sydney, New South Wales Australia; 3 Murdoch Children’s Research Institute, Parkville, Victoria Australia; 4 The Royal Children’s Hospital, Parkville, Victoria Australia; 5 Department of Paediatrics, The University of Melbourne, Parkville, Victoria Australia; 6 St Vincent’s Clinical School, UNSW Medicine, UNSW Sydney and Department of Clinical Pharmacology and Toxicology, St Vincent’s Hospital Sydney, Sydney, New South Wales Australia; 7 Department of Neurology, Austin Health, Heidelberg, Victoria Australia; 8 School of Nursing, Midwifery and Paramedicine, University of the Sunshine Coast, Maroochydore, Queensland Australia; 9 Children’s Health Queensland, Hospital and Health Service, University of Queensland, Brisbane, Queensland Australia; 10 IMPACCT, University of Technology Sydney, Sydney, New South Wales Australia; 11 Chris O’Brien Lifehouse Comprehensive Cancer Hospital, Camperdown, New South Wales Australia; 12 School of Medicine, University of Sydney, Sydney, New South Wales Australia; 13 NICM Health and Research Institute, Western Sydney University, Penrith, New South Wales Australia

**Keywords:** Cannabinoids, Human research ethics, Clinical trials, Cannabis medicines, Investigational medicinal product, Governance, Cannabis medicines research

## Abstract

With cannabis medicines now obtaining legal status in many international jurisdictions (generally on the authorisation of a medical professional), a rapid increase in consumer demand for access to cannabis as a therapeutic option in the treatment and management of a range of indications is being noted. Despite this accessibility, knowledge on optimal use is lacking. Further drug development and clinical trials at regulatory standards are necessary both if a better understanding of the efficacy of cannabis medicines, optimal product formulation and indication-specific dosing is needed and to ensure the broader quality and safety of cannabis medicines in the clinical setting.

To enable this, clinical, academic and public calls for the undertaking of rigorous clinical trials to establish an evidence base for the therapeutic use of cannabis medicines have been made internationally. While this commitment to undertake human studies with cannabis medicines is welcomed, it has highlighted unique challenges, notably in the review stages of ethics and governance. This often results in lengthy delays to approval by Human Research Ethics Committees (herein ‘HREC’, Australia’s nomenclature for Institutional Review Boards) and trial commencement. A principal concern in these cases is that in contrast to clinical trials using other more conventional pharmaceutical products, trials of cannabis medicines in humans often involve the use of an investigational product prior to some (or any) of the preclinical and pharmaceutical safety issues being established. This paucity of data around product safety, potential drug interactions, continuity of supply, shelf life and product storage results in apprehension by HRECs and governance bodies to endorse trials using cannabis medicines.

This manuscript draws from the experiences of Australian researchers and staff involved in clinical trials of cannabis medicines to describe some of the common difficulties that may be faced in the HREC approval process. It also presents practical advice aimed to assist researchers, HRECs and governance officers navigate this complex terrain. While the authors’ experiences are situated within the Australian setting, many of the barriers described are applicable within the international context and thus, the solutions that have been proposed are typically adaptive for use within other jurisdictions.

## Introduction

Like many countries, Australia has legalised the use of cannabis when used as a pharmaceutical-grade product prescribed by a medical professional for the therapeutic treatment of management of specific indications [1]. Unlike the standard pathway followed for other pharmaceutical products, a strong and public argument has been made by consumers and researchers for clinical trials to occur using cannabis medicines as investigational products in human studies prior to the preclinical and pharmaceutical safety issues being fully elucidated. Given this atypical approach, real-world ‘effectiveness’ clinical trials, including randomised controlled trials, are a means through which the risks of widespread use of off-label therapeutics and the collection of rigorous data to inform clinical practice can be balanced. However, understandably, this approach often raises seemingly complex issues for ethics and governance committees during the review and approval process.

A key concern of HRECs is that many of the studies of cannabis medicines are undertaken in the outpatient setting, involving self-medicating. Thus, issues such as potential use of a concomitant illicit product, adequacy of storage and dosing, measurement of toxicity, concomitant medication use and adequacy of follow-up are commonly raised. Further, the use of an unregistered product, such as cannabis medicines, in this relatively unsupervised setting introduces a novel set of additional issues for ethics and governance committees to overcome.

In 2019, a group consisting of Australian lead investigators of large investigator-initiated, Government or institutionally-funded studies; clinical trials officers; and regulatory staff came together to discuss common barriers and the lack of clear information available to guide the process of designing and undertaking clinical trials using cannabis medicines. Drawing on the current Australian Research Standards (such as the Australian National Guidelines, the *National Statement on Ethical Conduct in Human Research*) and the authors’ practical experience navigating these barriers this manuscript offers guidance as to how some of the key issues that arise when using cannabis medicines as investigational products can be remedied [2].

Through a series of discussions, six (6) common challenges were identified as the main barriers/issues faced when submitting clinical trials involving cannabis medicines to Human Research Ethics Committees (HREC) and regulatory authorities (such as Research Governance Offices (RGO) in Australia) for review and approval. These include (1) considerations when selecting cannabis medicines products, (2) accurate dosing and administration, (3) adverse events, (4) drug-drug interactions, (5) consent and (6) post-trial access to cannabis medicine products.

## Considerations when selecting cannabis medicines products in the trial setting

Sourcing a supply of cannabis medicines for use in clinical studies takes time, and the following should be considered and discussed in the ethics application when choosing a product for use:

- Does the product meet Good Manufacturing Practice (GMP) and current Federal and State regulations (such as the Therapeutic Goods (Standard for Medicinal Cannabis) (TGO 93) in Australia [3, 4])?
- Is there a guaranteed consistent supply available for the duration of the trial?
- Does the supplier of the product have knowledge of, and experience in the pharmaceutical environment (e.g. ability to provide an investigator’s brochure)?

Cannabis medicines are available in a variety of preparations with different concentrations of Cannabidiol (CBD), Delta-9-tetrahydrocannabinol (THC), other cannabinoids, varying terpene combinations, and maybe a whole plant product or a highly purified extract. Researchers and ethics committees both should be aware that one cannabis medicine product, even if available in the same dose, formulation or concentration, may have vastly different potency or purity to another. Cannabis medicine products may also have different absorption rates and pharmacokinetic profiles due to the variety of carrier oils, extraction methods and delivery systems available (such as vaporised, nanotechnology, oral vs. sublingual). The selection and formulation of the cannabis medicines product should, therefore, be clearly defined and explained in the research/study protocol.

Researchers need to be aware that cannabis medicines used in clinical trials, as with other registered products, must align with the existing framework for the use of unregistered/unapproved medications in clinical trials. This is done by Australia’s federal regulator, the Therapeutic Goods Administration (TGA) through their Clinical Trial Notification (CTN) scheme [5]. This scheme has the required elements to support the documented use of cannabis medicine products within a clinical trial in Australia [6]. Additionally, it is recommended that early consideration should be given to the classification or scheduling^1^ of the medicine and other prescribing regulations around such products, as this will have significant impacts on the logistics of a trial. In Australia, cannabis medicines are currently classified as either a Schedule 8—Controlled Drug or Schedule 4—Prescription Only Medicine, both requiring different national and state approval schemes for use [8]. For example, Schedule 8 medicines in the state of New South Wales, which includes cannabis medicine products containing THC, also requires State approval. Additional requirements may include specific features mandated by pharmaceutical regulations, such as the need for child-resistant openings, instructions for storage and use, labelling and movement and transportation of the medicine.

Researchers must confirm local requirements and consider the related implications, especially if planning a multi-site trial across borders. It can be helpful to have a specific section within the trial protocol which outlines these elements (referencing the relevant legislation), including issues around import/export and product transport, as well as the proposed methods of compliance for the clinical trial, to provide clarity for members of the ethics committee.

In the case of outpatient dosing, diversion can be limited by informing patients that reconciliation of their returned product will be performed upon return and encouraging patients to document drug consumption accurately, including any wastage.

An experienced clinical trial coordinator, collaborative research group or an accredited clinical trial pharmaceutical or contract research organisation can provide expertise in the import of products, labelling and packaging requirements and assist with meeting all required regulations. This is particularly important for an unregistered product, particularly when it may also be used with an unregistered device, such as vaporisers, which may also require approval from a regulatory body to be used in a clinical study. Seeking this support is strongly encouraged if the necessary skills are not contained within the supplier or the research team.

## Accurate dosing and administration

Cannabis medicines are unique when it comes to dosing and administration; apart from Nabiximols, there is currently no registered product information, which traditionally provides dosing schedules for new products. Dosing is individualised for each patient, with the clinicians and patients relying on titration to reach the optimum dose, if one exists, regardless of the cannabinoid content [9, 10]. In Australia, the Federal regulator recommends that starting doses should be low and increased over time until patients respond positively, or the negative effects outweigh the perceived benefits [11]. Product information dosing guidance has been developed in the interim to help ethics committees with this issue [9, 12]. Investigators can utilise a broad range of published data to outline the parameters they have used to inform the dosing schedule, which may include data in other populations, or from other cannabis medicines studies. To support informed ethical review, clarity around these data and decisions that have been made is an important part of the study rationale.

Additional factors that may affect dosing include the underlying condition being investigated, the type of product used, route of administration, dosage regimen, individual patient variation, possible development of tolerance, interaction with other medications and previous exposure to cannabis, either recreationally or medically [9].

As with all drugs, lower doses are less likely to be associated with adverse effects, possibly limiting therapeutic doses being achievable without toxicity, such as sedation without pain relief. To determine the efficacy of the cannabis medicine product for the patient and their medical condition, clinicians and patients need to work together to determine a personalised dosage escalation regimen, including starting dose and develop a plan for dose increments or decrements where the patient benefit is maximised, and adverse effects are minimised.

When selecting a cannabis medicine product for a clinical trial, the mode of administration is important, depending on whether a large exposure is needed, for example with chronic pain, where constant concentrations above a minimum are needed, or if a high maximum concentration is needed for a short time, e.g. for breakthrough pain. Rapid onset can be achieved by choosing an administration method bypassing metabolism in the liver, i.e. not oral. Examples include the commonly used inhaled (vaporised) route and oral mucosal, but developing routes for rapid absorption include transdermal, intranasal, buccal and sublingual routes. Researchers may also need to consider complexities such as using specific volumes and concentrations for doses and include and actual dose amount, such as milligram per day measured. Storage and administration devices such as droppers, vaporisers and non-pharmaceutical standard storage bottles must be checked for residual medicine and include specifications on the cleaning of delivery devices. Many of the above issues can potentially be eliminated by choosing a simpler, standardised route and a pharmaceutical-grade product and dispenser. One such option is encapsulating the medicine using preloaded vaporised dosing [13].

## Adverse events

Two elements of safety need to be considered in cannabis medicines trials; this is no different to the International Council for Harmonisation of Technical Requirements for Pharmaceuticals for Human Use (ICH) Good Clinical Practice (GCP) E6 (R2) requirements for clinical trials of other interventions. Firstly, the safety data which underpins the study design elements, such as participant population, dosing and expected adverse effects [14]. The second is to outline a safety monitoring approach, which is based on available data considering the population under study. The interpretation of safety data for cannabis medicines is complicated. While cannabis has been used by humans for millennia, details of the short- and long-term adverse reactions profile remains poorly understood. Overall, the known common adverse effects of cannabis medicines include sedation/somnolence, dizziness, anxiety, cognitive dysfunction, nausea, vomiting, diarrhoea, vertigo, increased or decreased appetite and dry mouth [15]. The adverse event profile of different formulations varies depending on the ratio of CBD: THC, the presence and amount of other cannabinoids and terpenes and the dose prescribed [16]. THC, particularly at higher doses, may be associated with treatment-emergent hallucinations, perceptual disturbances or paranoia [17].

ICH-GCP provides specific guidance on the procedures for assessing, classifying, documenting and reporting adverse events [2]. There is a lack of a standardised measure for assessing adverse events of cannabis medicines so all adverse events should be recorded and reported. Researchers may choose to use a measure such as the *Common Terminology Criteria for Adverse Events version 5* for describing adverse events [18, 19].

Blood tests may be conducted to assess for treatment-emergent abnormalities in haematology, biochemistry and liver functioning. Antiepileptic blood concentrations and concentrations of other drugs metabolised by the cytochrome P450 (CYP450) enzyme system may require monitoring depending on the study [20, 21].

In addition to routine biochemical and haematological monitoring, any unusual event, including large changes in heart rates and blood pressure, should be recorded both during the trial and for a specified duration after the trial has concluded. Furthermore, it should be noted that safety data from plant-derived cannabis products cannot be applied to synthetic cannabinoids, the safety of which needs to be considered independently.

## Drug-drug interactions

Currently, cannabis medicines used in clinical trials utilise either a single cannabinoid extract (THC or CBD) or a combination of these two cannabinoids in varying ratios, and as part of a whole plant product or as combined extracts, with or without added minor cannabinoids or terpene extracts. The pharmacokinetics and pharmacodynamics of most analogue cannabinoids are not known and little work has been undertaken on the stereoisomeric forms of the cannabinoids or pharmacogenomic studies on their metabolism [22].

THC and CBD are metabolised by the CYPP450 system. The CYP system is also a common site for drug-drug interactions, which have been responsible for many deaths in Australia [23]. Inhibition by drugs such as ketoconazole and clarithromycin significantly increase concentrations of THC and CBD, while inducers such as rifampicin, carbamazepine and St John’s Wort lower THC and CBD concentrations. Other, pharmacodynamic (PD) interactions may occur at the level of the endocannabinoid receptor but are not yet known, including G-protein-coupled receptors (GPCR) interactions and common binding of cannabinoids to mu, dopamine, serotonin and similar receptors [24].

As per standard clinical trial practice, complete details of any co-administered medications, alcohol intake, tobacco smoking and complementary medications should be recorded, and dosing of a cannabis medicine product altered as medication changes. Adherence to GCP guidelines requires that the mode of delivery, duration of cannabis medicines therapy and previous exposure to cannabis are documented in the evaluation of any therapeutic effect. Similarly, this information needs to be reported in accordance with good pharmacovigilance practice in the recording of adverse drug reactions.

## Consent

There has been a strong consumer demand for cannabis medicines [25], particularly in the setting of life-limiting conditions and symptom control. The population is generally comprised of vulnerable people, such as children with epilepsy, patients moving towards the palliative care phase of their illness and people living with mental health issues, or drug and alcohol dependence [26]. Often, these vulnerable populations are willing to ‘try anything’ to alleviate difficult to manage symptoms.

In line with the Australian National Guidelines, participation in cannabis medicines trials, as with any clinical trial, must be voluntary and based upon enough information, and with the ability to withdraw consent and involvement in research at any time without prejudice [2, 27]. An individual’s capacity to consent must be assessed, and it is important to note that a participant’s capacity may fluctuate with time and situation during trials using cannabis medicines due to sedative effects [28]. While it is not a requirement in Australia that consent is routinely witnessed [2], we recommend that participant consent should be witnessed in cannabis medicines trials to ensure participant safety and self-determination. Participants must also be advised that they will be unable to drive or operate heavy machinery while taking a cannabis medicine product containing THC.

## Post-trial access

Post-trial access is an ethical obligation should the study drug prove safe and effective and must be considered during the development of the protocol. Researchers should confirm ongoing access pathways and communicate this to participants at the time of consent [29]. Consideration should also be given to the most appropriate alternative cannabis medicine product in the event of supply issues. Due to the botanical nature of the source of the study drug and variation in base excipients between manufacturers, it may be difficult to identify an alternate product to the specific medication used in a researcher’s clinical trial.

To ensure ongoing clinical care and safety monitoring of a participant, open-label extension of a clinical trial is common, with the treatment provided at no cost and the benefit of continued data collection in a managed access programme [30]. The CARE NSW trial in Australia is an example of such a trial to adopt this mechanism in the advanced cancer population; however, this is uncommon due to cost, sponsor unwillingness and logistical issues, such as coordination of access by research staff, who may be employed only for the period of the trial. Open-label extensions are not an ethical requirement; however, patients must be informed that medication supply will not be available at the end of the study [31].

The alternative that can be included in a study protocol is to utilise the existing framework for prescribing non-registered medicines. In Australia, approved cannabis medicine products which meet TGO93 may be prescribed post-trial at the direct cost of the patient through Australia’s TGA special access schemes (SAS) or by their doctor obtaining personal authority to be a registered prescriber of cannabis medicines [32]. It is, however, important to highlight the cost burden of this to participants in advance.

Availability of and access to cannabis medicines for participants post-trial must, therefore, be confirmed during protocol development, and arrangements should be clearly communicated in the Protocol and Patient Information Sheet and Consent Form (PICF).

## Conclusion

Notwithstanding the need to adhere to the National, Federal and State guidelines, recently clinicians, public health professionals and academics are concerned about the barriers arising through the ethics review process, often due to the complexity for committees of navigating large clinical trials using (often unregistered) cannabis medicines. While cannabis medicines are a relatively new and promising therapy, clinical trials need to be undertaken to understand how to demonstrate their efficacy and safety in different conditions. Thus, it is likely that ethics and governance committees will receive an increasing number of research protocols involving studies of cannabis medicines for review. However, because cannabis is also an illicit substance when used outside of regulated medical settings, it is imperative to educate staff working in the ethics and governance about the major differences between illicit cannabis and cannabis medicines. Education and discussion for all key stakeholders to address the current perceived risks, including product safety, regulations, storage and supply will ease concerns from ethics committees around such trials.

## Data Availability

Not applicable

## Abbreviations

CARE NSW: Cannabinoids for Symptom Control in Advanced Cancer, an Open-Label Prospective Clinical Trial in New South Wales (NSW)
CBD: Cannabidiol
CTN: Clinical Trial Notification
CYP450: Cytochrome P450
GPCR: G-protein-coupled receptors
GMP: Good Manufacturing Practice
HREC: Human Research Ethics Committee
ICH-GCP: International Council for Harmonisation of Technical Requirements for Pharmaceuticals for Human Use Good Clinical Practice
PD: Pharmacodynamic
PICF: Patient Information Sheet & Consent Form
RGO: Research Governance Office
SAS: Special Access Scheme
TGA: Australia’s Therapeutic Goods Administration
TGO 93: Therapeutic Goods (Standard for Medicinal Cannabis)
THC: Delta-9-tetrahydrocannabinol

## Footnotes

1 Under the Australian regulatory system overseen by the Therapeutics Goods Administration (TGA), medicines and poisons are scheduled according to the level of regulatory oversight required to protect public health and safety [7]. Therapeutic Goods Administration. Scheduling of medicines & poisons [Internet] 2016 [cited 07 August 2020]. Available from: https://www.tga.gov.au/scheduling-medicines-poisons
